# The University of Padua salivary-based SARS-CoV-2 surveillance program minimized viral transmission during the second and third pandemic wave

**DOI:** 10.1186/s12916-022-02297-1

**Published:** 2022-02-23

**Authors:** Daniela Basso, Ada Aita, Filippo Navaglia, Paola Mason, Stefania Moz, Alessio Pinato, Barbara Melloni, Luca Iannelli, Andrea Padoan, Chiara Cosma, Angelo Moretto, Alberto Scuttari, Daniela Mapelli, Rosario Rizzuto, Mario Plebani

**Affiliations:** 1 Department of Medicine – DIMED, Laboratory Medicine, University Hospital of Padua, Via Giustiniani 2, 35128 Padua, Italy; 2 Department of Laboratory Medicine, University-Hospital of Padua, Padua, Italy; 3 Department of Cardiac, Thoracic, Vascular Sciences and Public Health, Occupational Health Unit, University of Padua, Padua, Italy; 4 Environment and Safety Office, University of Padua, Padua, Italy; 5 Software Development Office - IT Service, University of Padua, Padua, Italy; 6 General Director of the University of Padua, Padua, Italy; 7 Pro-rector for teaching of the University of Padua, Padua, Italy; 8 Chancellor of the University of Padua, Padua, Italy

**Keywords:** SARS-CoV-2 spread, Active surveillance, University employees, Salivary testing

## Abstract

**Background:**

The active surveillance of students is proposed as an effective strategy to contain SARS-CoV-2 spread and prevent schools’ closure. Saliva for molecular testing is as sensitive as naso-pharyngeal swab (NPS), self-collected and well accepted by participants. This prospective study aimed to verify whether the active surveillance of the Padua University employees by molecular testing of self-collected saliva is an effective and affordable strategy for limiting SARS-CoV-2 spread.

**Methods:**

A surveillance program based on self-collection of saliva every 2 weeks (October 2020–June 2021) was conducted. Among 8183 employees of the Padua University, a total of 6284 subjects voluntarily took part in the program. Eight collection points guaranteed the daily distribution and collection of barcoded salivary collection devices, which were delivered to the laboratory by a transport service for molecular testing. Quarantine of positive cases and contact tracing were promptly activated.

**Results:**

Among 6284 subjects, 206 individuals were SARS-CoV-2 positive (99 by salivary testing; 107 by NPS performed for contact tracing or symptoms). The cumulative SARS-CoV-2 incidence in this cohort was 3.1%, significantly lower than that of employees not in surveillance (8.0%), in Padua (7.1%) and in the Veneto region (7.2%). Employees with positive saliva results were asymptomatic or had mild symptoms. The levels of serum antibodies after 3 months from the infection were correlated with age and Ct values, being higher in older subjects with greater viral loads.

**Conclusions:**

Salivary-based surveillance with contact tracing effectively allowed to limit SARS-CoV-2 contagion, also in a population with a high incidence.

## Background

SARS-CoV-2 vaccination is considered the most effective strategy for combatting the COVID-19 pandemic, allowing countries to fully restart their activities without the need of further lockdown [1, 2]. However, the transition period between the start and the end of vaccination programs might take several months, it is not yet known exactly how long vaccinations are protective against COVID-19, or whether they protect individuals against emerging variants [2]. Therefore, effective surveillance and contact tracing policies are of crucial importance in containing viral spread while allowing the opening of social, educational, and productive activities [1]. The passive strategy of surveillance (i.e., testing of symptomatic individuals and contact tracing of SARS-CoV-2-positive subjects) [3] adopted in many parts of the world during the first, second, and third waves of the pandemic is frail, since it fails to trace asymptomatic and pre-symptomatic infected subjects, who may disseminate the infection as widely, if not more so, than symptomatic subjects [4]. Active strategies designed to surveil large groups of asymptomatic individuals have therefore been proposed in order to limit contagion while allowing activities to be opened up.

Effective, acceptable, and affordable programs should be proposed for the active surveillance of large series of subjects, such as school students, teachers, or employees of small-medium and large enterprises [5]. For an effective strategy, the most accurate possible method for SARS-CoV-2 detection should be chosen. Currently, molecular testing of naso-pharyngeal swabs (NPS) is recommended as the gold standard method, although its sensitivity and specificity are not absolute [6, 7]. As an alternative, in the case of limited resources and/or the urgent clinical need for test results, rapid antigen tests using nasal or NPS are feasible if their sensitivity is higher than 80% and specificity higher than 97%, following specific timing of repeated testing [8]. The enormous variety of rapid antigen tests includes several lateral flow immunochromatographic and a few immunofluorescent assays. These tests overall appear to have a limited sensitivity, especially when viral loads are not high (i.e., rRT-PCR Ct values above 25) [9–13]. This limitation does not support rapid antigen testing in active surveillance, and the use of the gold standard NPS molecular testing in large series calls for specific medical services and personnel trained in sample taking. Self-collected saliva-based testing, which reduces costs by 25 to 30% in a context of active surveillance [3, 14], is tolerated better than NPS, thus maximizing compliance. Furthermore, saliva testing using molecular methods, real-time reverse transcription polymerase chain reaction (rRT-PCR) or quantitative loop-mediated isothermal amplification (QLAMP), is highly accurate: several studies, including those from our group, have demonstrated a pooled sensitivity of 83 to 88% against a specificity approaching 99% [6, 7, 15, 16]. Self-collected saliva for SARS-CoV-2 molecular testing might therefore be effective and acceptable for active surveillance [17], pooled testing being a cost-effective strategy for repeat and routine surveillance [3, 14], although it remains to be elucidated whether it is affordable. In ascertaining affordability, it is important to include all steps of the total testing process, namely (1) the pre-analytical phase, which includes scheduling, sampling, and transport to the laboratory; (2) the analytical laboratory phase, which includes sample preparation, nucleic acid extraction, and rRT-PCR; and (3) the post-analytical phase, which includes the validation of analytical results, their communication, and further action (contact tracing of positive cases). Last, but not least, a careful consideration should be made as to whether or not the active screening strategy is cost-effective.

The aim of the present prospective study was to verify whether the active surveillance of Padua University employees by molecular testing of self-collected saliva is effective, acceptable, and affordable for limiting SARS-CoV-2 spread.

## Methods

### Study design and participants

**Fig. 1:**
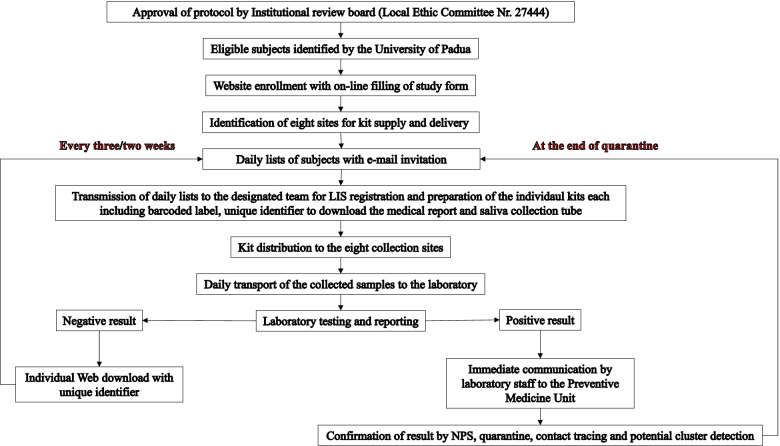
Active surveillance program: study design and workflow

This prospective study, conducted from October 8, 2020, to June 30, 2021, was based on saliva molecular testing performed every 3 weeks for SARS-CoV-2 surveillance of the employees of the University of Padua, who participated on a voluntary basis. The time interval for repeated testing was shortened to 2 weeks in November 2020 due to the increasing incidence of the infection in our geographic area, the Veneto region. This time interval was then maintained until the end of the study period. Figure 1 shows the study workflow. In September 2020, the University of Padua employees were invited to participate in the study by announcement and personal e-mail invitation. The invited employees included the following categories: full, associate, and adjunct professors; researchers; scholarship students; postdocs; PhD students; office workers; and technicians. Employees in the medical sector (*n*=2552) were not invited because they participated in the surveillance program of the University Hospital of Padua based on regular NPS molecular testing according to the National regulation. Temporary staff members offering their services to the University (e.g., receptionists, part-time tutors) were also invited. For participants, a video tutorial providing instructions for saliva collection, sample delivery and report downloading, and FAQ were prepared and published on the University website. All participants were assigned collection days, which were spread 3 weeks apart from each other. This resulted in about 400 people being tested per day. The University identified eight collection points where, the day before sampling, all employees were given Salivette devices (Sarstedt AG & Co, Germany), each with its barcode and individual code for access to the test result, which could be downloaded from the Laboratory website. On the sampling day, between 8 and 10 am, employees returned the Salivette in dedicated safety boxes to the same point. Boxes were then collected by a transport service (Plurima S.p.A., Milano, Italy) for transportation to the laboratory.

### Sample collection and analysis

Saliva, collected using a standardized procedure by a barcoded Salivette, was obtained after centrifugation at 4000 *g* × 5 min. Pairs of two salivary samples were pooled using 100 μl of each sample. The 200-μl pool was added with 100 μl MagNA Pure 96 External lysis buffer (Roche Diagnostics, USA) using the liquid handler MagNA Starlet (Hamilton, Switzerland). Nucleic acid was extracted by automated Magna Pure 96 Instrument (Roche Diagnostics), with complete informatic sample tracing. rRT-PCR of Orf1ab, N, and S SARS-CoV-2 genes was made using the TaqPath COVID-19 RT-PCR kit on QuantStudio 5 instruments (Thermo Fisher, USA). In the case of any positive saliva pool result, the two individual saliva samples were immediately analyzed singly. Turn-around-time ranged from 12 to 24 h, samples being classified as positive when at least two analyzed genes gave a positive amplification curve, with a cycle threshold (Ct) value of less than 36. The laboratory medical staff promptly communicated all positive results to the Preventive Medicine and to the Environment and Safety office. The Preventive Medicine occupational physician immediately contacted all positive subjects by phone and invited them to undergo molecular NPS to confirm the finding. The same occupational physician activated contact tracing and quarantine policies for individuals who came into contact with the confirmed SARS-CoV-2-positive person. All SARS-CoV-2-positive University of Padua employees, whether or not under the surveillance program, were invited after 3 and 6 months from infection to undergo antibody testing. In the 3 to 6-month time frame, the vaccination campaign of the University of Padua was launched and 37/65 subjects enrolled at 6 months received one dose of Astra Zeneca – Oxford University ChAdOx1/AZD1222 and 17/65, one dose of PfizerBiontech BNT162b2 vaccine. Serum anti-SARS-CoV-2 antibodies were measured by chemiluminescent immunoassay (CLIA), which identified IgG anti-RDB (Snibe Diagnostics, New Industries Biomedical Engineering Co., Shenzhen, China) on the automated platform MAGLUMI™ 2000 Plus (Snibe Diagnostics) [18].

Population-related epidemiological data were retrieved from the Italian database (https://mappe.protezionecivile.gov.it/it/mappe-emergenze/mappe-coronavirus/situazione-desktop).

### Statistical analysis

The statistical analysis included all data collected from 8 October 2020 to 13 May 2021. Two-sample Wilcoxon rank-sum test, multiple linear regression analysis, chi-square test, and the Wilcoxon matched-pairs signed rank test were performed using Stata software 13.1 (StataCorp, 4905 Lakeway Drive, College Station, TX, 77845 USA).

## Results

### Participation and compliance to the study program

**Fig. 2:**
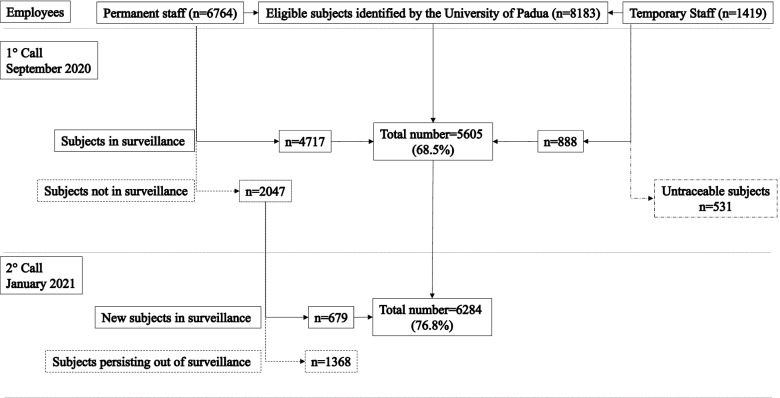
Flow chart diagram reporting the number of subjects under surveillance and not in surveillance involved in the study. The 531 non-permanent staff subjects who did not participate in the study were excluded since, as non-permanent, their medical records were not always available to the Preventive Medicine of the University of Padua

In September 2020, a total of 8183 subjects, including 6764 employees and 1419 temporary staff members of the University of Padua, were invited to participate to the study (Fig. 2). In the period October–November 2020, 5580/8183 subjects (68%) started the program providing the first salivary sample (Table 1). In December 2020, only 25 subjects were newly enrolled. In order to further increase the percentage of employees under surveillance, a second invitation was launched in January 2021 leading to 556 new subjects starting the program between January and March 2021. Only a small number of employees (*n*=123) provided their first salivary sample at the end of the study in April–May 2021. The mean number of employees who participated in the study every month from October 2020 to May 2021 was 4790, ranging from a minimum of 3965 subjects in May 2021 to a maximum of 5447 in November 2020.

**Table 1:** Number of new subjects entering the surveillance program from October 2020 to May 2021

Month	New subjects entering the surveillance program (number)	Gender (number)		Age (mean±standard deviation)		
		Female	Male	Total	Female	Male
October 2020	4101	2117	1984	44**±**12.032	43**±**11.524	44**±**12.54
November 2020	1479	737	742	40**±**13.04	39**±**12.64	40**±**13.38
December 2020	25	10	15	47**±**14.61	41**±**14.55	50**±**13.84
January 2021	23	7	16	39**±**13.41	36**±**11.16	41**±**14.27
February 2021	240	112	128	38**±**12.19	37**±**11.57	39**±**12.71
March 2021	293	170	123	36**±**13	34**±**11.30	39**±**14.68
April 2021	110	31	79	45**±**12.41	44**±**14.40	46**±**11.58
May 2021	13	8	5	32**±**11.97	29**±**10.08	37**±**14.33

As illustrated in Fig. 2, two cohorts of subjects were identified: employees under surveillance and employees not in surveillance. The employees under surveillance included the subjects who participated in the study, 5605 subjects from October to December 2020 and 6284 subjects from January to May 2021. The employees not in surveillance included the remaining University permanent staff which clinical status was traced by the Preventive Medicine of the University of Padua, 2047 subjects from October to December 2020 and 1368 subjects from January to May 2021.

Table 2 shows the number of repeated tests (range, 1–15) that employees underwent.

**Table 2:** Number of repeated tests that employees underwent during an active surveillance program

Repeated tests (number)	Employees (number)
1	259
2	297
3	255
4	372
5	285
6	140
7	199
8	273
9	396
10	745
11	988
12	1811
13	262
15	2

We considered as compliant those subjects that repeated testing at least 9 times, which takes into consideration the following: (1) At the beginning of the study, in October 2020, a 3-week interval was planned and changed to 2 weeks in mid-November 2020; (2) during Christmas and Easter holidays, monitoring was stopped; (3) an expected reduction of testing in May 2021, due to ending of teaching activities at the University associated with very low incidence in the general population. In the majority of cases (4204/6284, 67%) the enrolled subjects demonstrated a high level of compliance, undergoing at least nine replicated salivary tests, thus completing all required testing rounds. Few subjects underwent three or fewer tests (811/6284, 13%).

### Salivary molecular testing: results and actions

Every week 1778 ± 374 (mean ± standard deviation) salivary samples were analyzed, for a total of 55,998 samples received from October 2020 to May 2021. Among them, 1415 (2.5%) were rejected because of incorrect use of the salivette device (*n*=578), incorrect labeling (*n*=836), or insufficient volume (*n*=1).

**Fig. 3:**
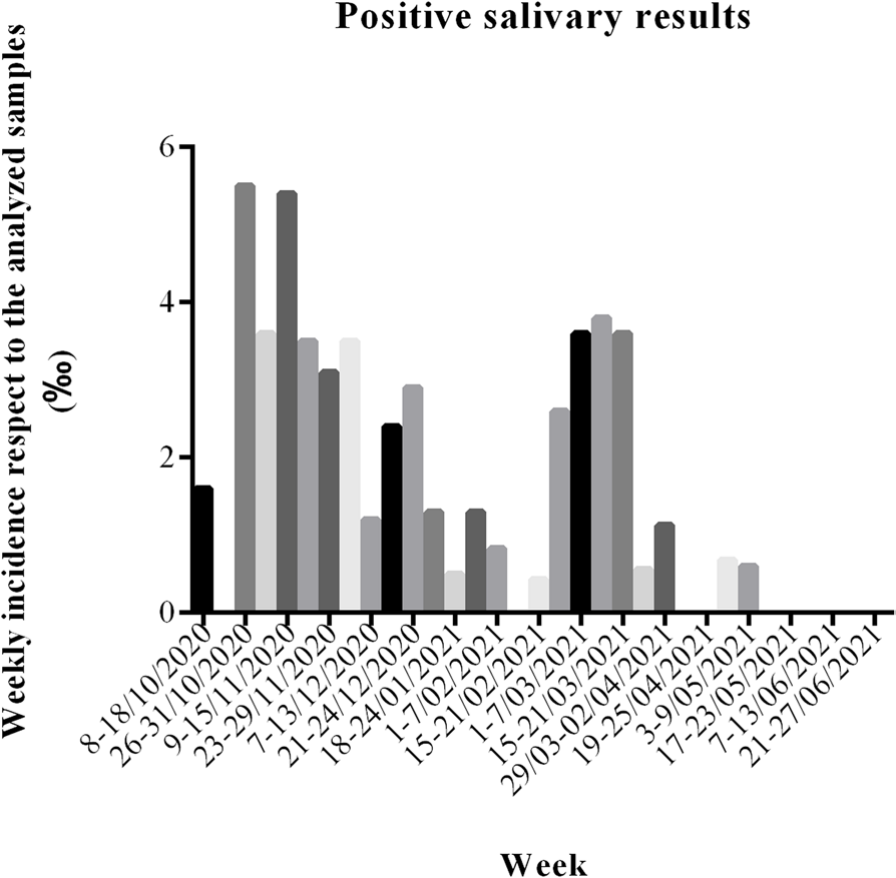
Weekly incidence of positive salivary results against the overall number of tested samples

Figure 3 reports the weekly incidence of positive salivary results against the overall number of tested samples. The incidence, which peaked in November 2020, progressively declined until January, to increase again in March 2021. As from mid-May, until the end of the study period, no more positive cases were identified.

**Fig. 4:**
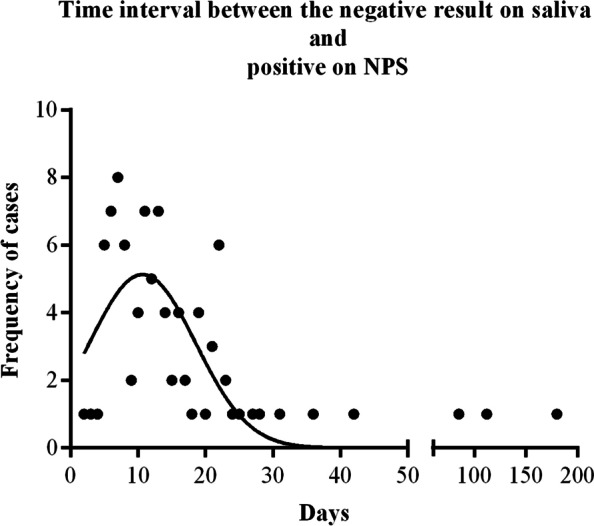
Time interval between negative salivary test and SARS-CoV-2 detection by NPS, because of symptoms or contact tracing occurred among employees under surveillance

SARS-CoV-2 infection was diagnosed by saliva testing in an overall number of 99 subjects, three of whom remained positive for more than 2 weeks; this resulted in an overall number of 102 positive saliva test results. All subjects with positive salivary SARS-CoV-2 results underwent NPS molecular testing within 24 h from saliva sampling, positivity being confirmed in 94/99 cases. Two of the five subjects with negative NPS findings developed typical COVID-19 symptoms (fever and anosmia, respectively). SARS-CoV-2 infection was identified in an additional 107 surveilled employees who underwent NPS for contact tracing (*n*=1) or because of symptoms (*n*=106). Thirteen of these 107 SARS-CoV-2-positive subjects started salivary-based surveillance after a positive NPS result. For the remaining 94 subjects, the time interval between the last salivary test and the positive NPS result (Fig. 4) was 17 ± 22 days (mean ± standard deviation) with a median of 17 days (interquartile range (IQR) 8–19 days). This time interval was significantly different from that (i.e., the time lapse between a positive saliva result and a previous negative saliva result) recorded in the group of 99 positive subjects identified with salivary testing (16 ± 10 days, mean ± standard deviation; median 16 days; IQR: 14–20 days) (two-sample Wilcoxon rank-sum test: *z* = 2.389, *p* = 0.017). In the vast majority (79/94, 84%) of subjects positive at NPS undertaken for symptom onset after a negative salivary test, the time lapse between salivary and NPS testing was longer than 6 days. Moreover, a significant difference was found between the overall number of repeated saliva tests made by SARS-CoV-2-positive subjects identified by salivary test (mean ± standard deviation = 8 ± 4; median=9; IQR = 5–11) and those identified by NPS (mean ± standard deviation = 7 ± 4; median = 8; IQR = 4–10) (chi-squared = 7.134, *p* = 0.0076). SARS-CoV-2-positive subjects were not excluded from surveillance, but allowed to participate after the end of quarantine and negative NPS molecular testing.

**Fig. 5:**
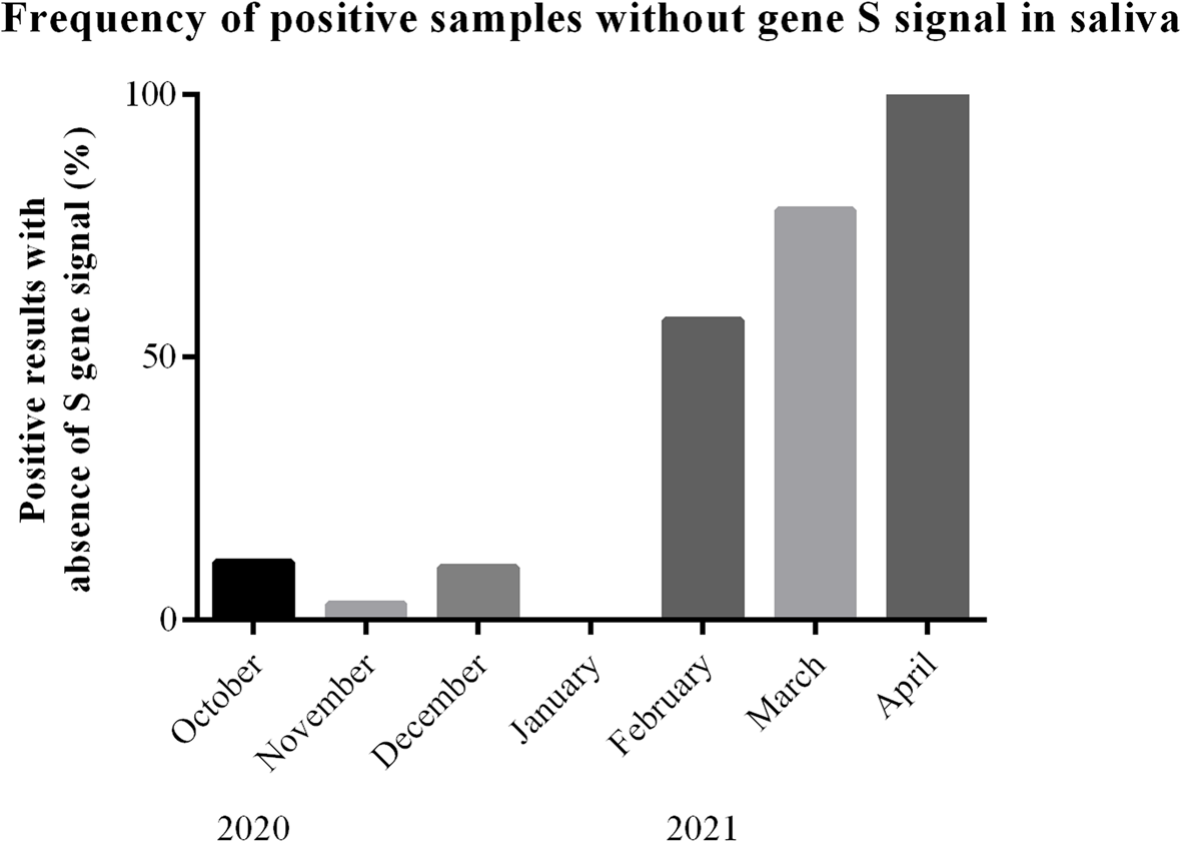
Frequency of positive samples without the S gene signal during the active surveillance program

Saliva test results were classified as positive if at least two out of the three genes evaluated (Orf1ab, N, and S) were positive. Orf1ab and N were both positive in all 99 positive cases, while the S gene was positive in 72/99 positive cases. The frequency of positive samples without the S gene signal was low in 2020 but increased exponentially as from February 2021 to reach 100% in April 2021 (Fig. 5). As communicated by Thermo Fisher on 1 February 2021, the TaqPath COVID-19 test will result in S gene dropout in samples with a variant carrying the 69-70del mutation [19].

### SARS-CoV-2 incidence in surveilled and non-surveilled employees

**Fig. 6:**
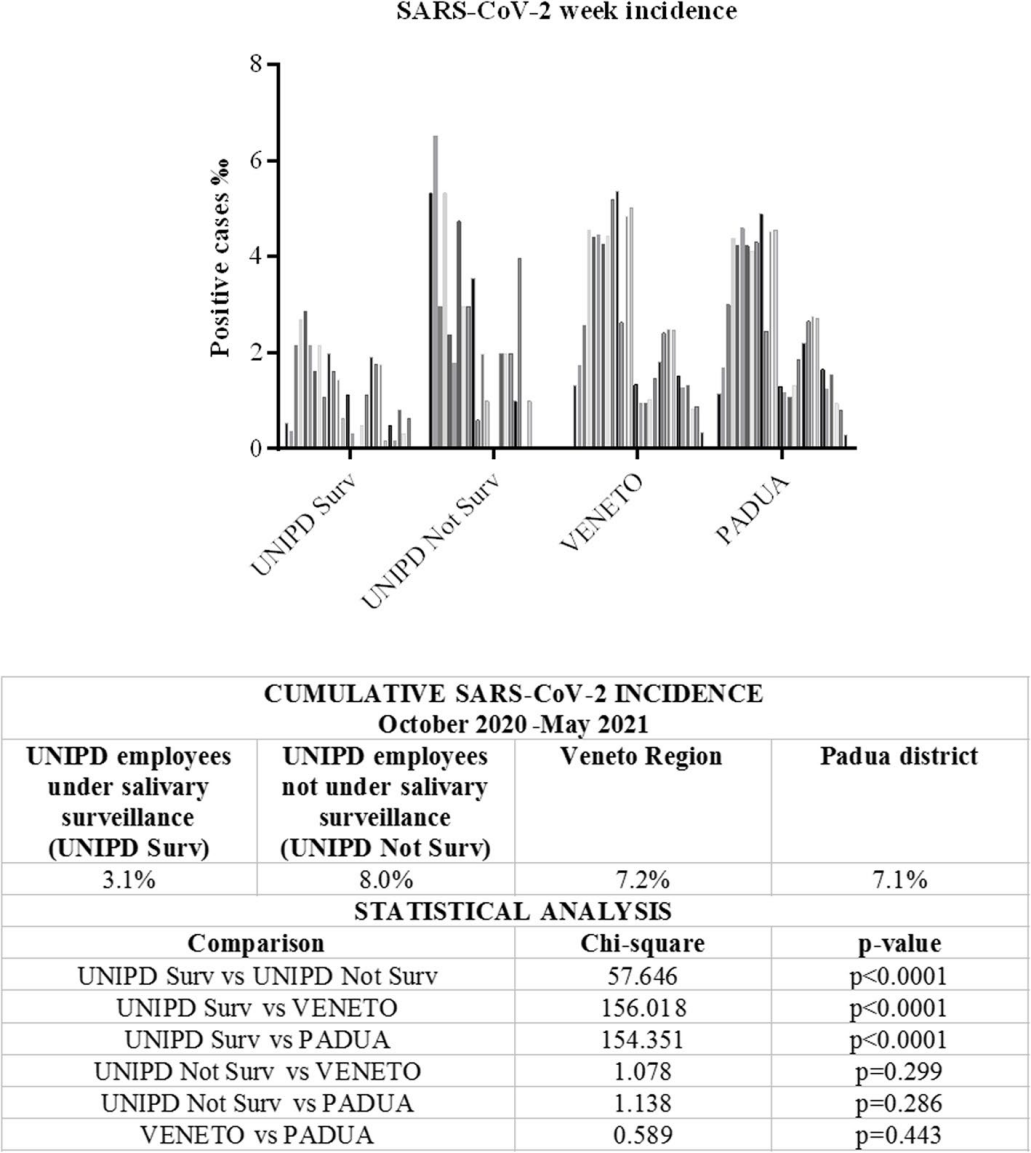
Weekly incidence of SARS-CoV-2 during the active surveillance program. The weekly incidence found in the two cohorts of employees (under surveillance or not) was shown in comparison with the population incidence for the Veneto region and the district of Padua

The weekly incidence of SARS-CoV-2-positive cases observed in the two cohorts of employees, under surveillance or not, was compared with the population incidence for the Veneto region and the district of Padua; the results are shown in Fig. 6. The cumulative frequency of positive cases among employees under surveillance (3.1%) was significantly lower than in non-surveilled employees (8.0%) in the Padua district (7.1%; 933,700 inhabitants) and in the surrounding Veneto region (7.2%; 4,879,133 inhabitants).

Among the series of SARS-CoV-2-positive employees, which included only one subject requiring hospitalization, the clinical symptoms were recorded from 162 subjects by phone interviews. The occupational physician asked participants whether or not they had symptoms immediately before, or soon after, salivary or a positive NPS result. Eighty-four cases were in the series identified by salivary testing and 78 by NPS. The frequency of symptoms, and the statistical analysis of data, is reported in Table 3. The vast majority of SARS-CoV-2 subjects identified by positive saliva test results were asymptomatic or presented few, mild symptoms. Fever, ageusia, and gastrointestinal symptoms were more frequently recorded among positive subjects identified by NPS.

**Table 3:** Frequency of symptoms referred by SARS-CoV-2-positive employees of the University of Padua. Positive subjects comprised 84 individuals identified by surveillance salivary testing and 78 individuals who directly underwent NPS for suspected clinical manifestations

	Saliva-based diagnosis (*n*=84)	NPS-based diagnosis (*n*=78)	Chi-square	*p*-value
Fever	26 (31%)	45 (58%)	11.7465	*p* = 0.001
Anosmia	12 (14%)	15 (19%)	0.7121	*p* = 0.399
Ageusia	7 (8%)	15 (19%)	4.0925	*p* = 0.043
Asthenia	20 (24%)	25 (32%)	1.3694	*p* = 0.242
Cough	17 (20%)	21 (27%)	1.0067	*p* = 0.316
Headache	11 (13%)	17 (22%)	2.1411	*p* = 0.143
Gastrointestinal symptoms	0	4 (5%)	4.4167	*p* = 0.036
Arthralgia	5 (6%)	9 (12%)	1.5985	*p* = 0.206
Myalgia	8 (10%)	13 (17%)	1.8289	*p* = 0.176
Dyspnea	2 (2%)	2 (3%)	0.0056	*p* = 0.940
Rhinitis	24 (29%)	21 (27%)	0.0548	*p* = 0.815
Sore throat	12 (15%)	11 (14%)	0.0092	*p* = 0.924

### Antibody testing: response to disease and to vaccines

**Fig. 7:**
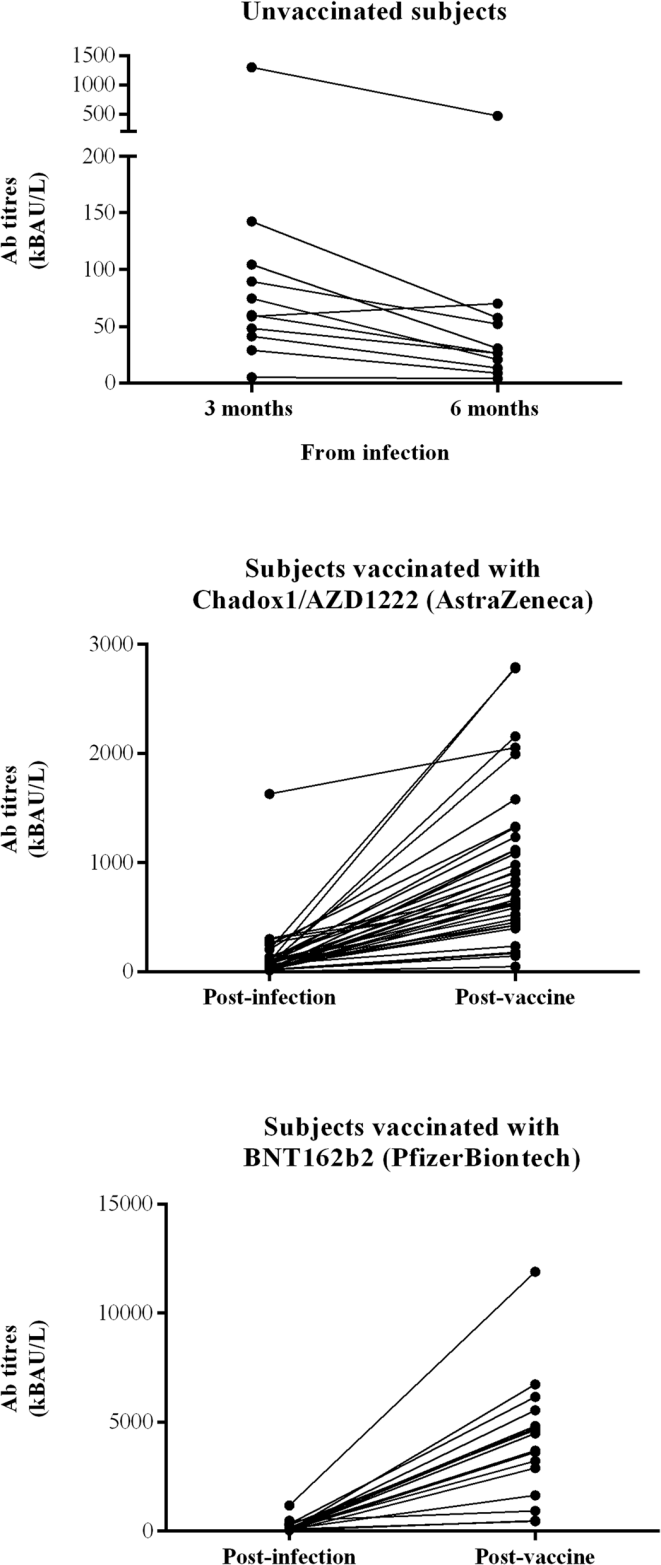
Variations of serum antibody titres (anti-RBD IgG) at 3 and 6 months after SARS-CoV-2 infection. The upper panel shows the results of subjects who remained unvaccinated. The middle and lower panels show the results obtained from subjects that received Chadox1/AZD1222 or BNT162b2 vaccine respectively. Post-infection antibody levels are those found after 3 months from SARS-CoV-2 infection. Post-vaccine antibody levels are those found after 6 months from SARS-CoV-2 infection and 3 months after vaccination

Those employees who had SARS-CoV-2 infection in the period October–December 2020, whether under surveillance or not, were asked to perform antibody testing after 3 and 6 months from the diagnosis. At 3 months (January–March 2021), 104 subjects were enrolled. Of these, 65 performed antibody testing also at 6 months. At 3 months, none of the 104 enrolled subjects was vaccinated, while part of them received one vaccine dose in the time frame April–June 2021, by giving their adhesion to the vaccination campaign of the University of Padua. Among the 65 subjects who performed antibody testing at 6 months, 11 remained not vaccinated, 37 received Chadox1/AZD1222, and the remaining 17 had BNT162b2. Three months after diagnosis of infection, antibody levels ranged from 0.1 to 376 kBAU/L with a median of 16 and an IQR of 7–29 kBAU/L. Figure 7 shows the paired titer variations at 3 and 6 months in unvaccinated subjects, in Chadox1/AZD1222, and in BNT162b2-vaccinated subjects. A significant decline in unvaccinated (*p*=0.0029) and a significant increase in Chadox1/AZD1222 and BNT162b2-vaccinated subjects (*p*<0.0001) was found (Wilcoxon matched-pairs signed rank test).

Multivariate regression analyses were performed considering antibody titers at 3 and 6 months as dependent variables. At 3 months, age, gender, presence or absence of symptoms, and Orf1ab Ct values at diagnosis were included as predictor variables (Table 4). At 6 months, age, gender, antibody titers at 3 months, and type of vaccine were included among predictors (Table 5).

**Table 4:** Serum antibody at 3 months after SARS-CoV-2 diagnosis was correlated with age. Multiple linear regression analysis was performed considering serum antibody titers at 3 months after SARS-CoV-2 diagnosis as a dependent variable. Predictors entered in the analysis were: age, gender, presence or absence of symptoms, and *Orf1ab* Ct values at diagnosis

	Coefficient	Standard error	***t***	***P***	95% confidence interval
**Age**	9.356	3.113	3.01	0.005	3.049 to 15.663
**Gender**	−112.246	82.533	−1.36	0.182	−279.4747 to 54.982
**Symptoms at diagnosis**	110.424	72.283	1.53	0.135	−36.03577 to 256.883
***Orf1ab*** **Ct values at diagnosis**	−19.887	7.602	-2.62	0.013	−35.28907 to −4.484
**Cons**	380.219	298.291	1.27	0.210	−224.175 to 984.614

**Table 5:** Serum antibody at 6 months after SARS-CoV-2 diagnosis was correlated vaccine type. Multiple linear regression analysis was performed considering serum antibody titers at 6 months after SARS-CoV-2 diagnosis as a dependent variable. Predictors entered in the analysis were: age, gender, antibody titers at 3 months and type of vaccine

	Coefficient	Standard error	***t***	***P***	95% confidence interval
**Age**	21.844	17.821	1.23	0.226	−13.968 to 57.656
**Gender**	334.568	414.464	0.81	0.423	−498.329 to 1167.465
**Antibody titers at three months from SARS-CoV-2 infection**	2.311	0.789	2.93	0.005	0.726 to 3.897
**Type of vaccine**	3017.549	439.968	6.86	0.000	2133.400 to 3901.697
**Cons**	−3805.621	1131.542	−3.36	0.002	−6079.539 to −1531.702

Infection-induced antibody levels (3 months) were dependent on age and Orf1ab Ct values, while vaccination-induced antibody levels (6 months) were dependent on previous antibody levels and vaccine type.

## Discussion

The vaccination campaign brought about a significant improvement in the containment of the SARS-CoV-2 pandemic, with a dramatic limitation in the number of hospitalizations during the fourth wave. Despite the high percentage of people that have been, or soon will be, vaccinated, millions of individuals still have no SARS-CoV-2 protection immunity, and are therefore vulnerable. In the near future, a parallel strategy based on large-scale vaccination on the one hand and SARS-CoV-2 testing and contact tracing on the other will probably be the key to containing viral diffusion while reopening all productive and education activities, also in view of the potential vaccine escape of emerging variants [20]. The findings made in the present large cohort prospective study demonstrate that salivary-based surveillance with contact tracing is an effective measure that limits the spread of infection, also in a population with a high incidence of the disease.

The study, started in October 2020, involved University of Padua employees. A large number of subjects took part in the program and, importantly, numerous subjects completed the 9-months periodic surveillance period. This high compliance may have been underpinned by effective communication from the University to its employees, by the overall consciousness that individual and population health are strictly dependent on each other, by individual fear and, last but not least, by the user-friendly, non-invasive nature of sampling saliva, the preferred fluid for COVID-19 diagnostics among healthcare workers [21]. We chose saliva molecular testing as an alternative to NPS, first because of its high sensitivity and specificity, comparable to those of NPS [7, 22], and second because its collection is easy to achieve, and well tolerated [21]. The different saliva collection methods proposed include general spitting and drooling, but these options might impact on results, spit reportedly being correlated with a lower sensitivity [6, 22]. In our study, samples were collected in standardized conditions after overnight fasting without tooth cleaning, and by means of a standardized device previously demonstrated by us and others to allow sensitive and reproducible results without incurring the risk of unsuitable samples [16, 23–25].

The findings made in this prospective study confirm that results obtained with molecular SARS-CoV-2 saliva testing are comparable to those with NPS, 95% positive saliva results being confirmed at NPS. The finding that five subjects had positive saliva results, but negative NPS, which was not unexpected, might reflect SARS-CoV-2 dynamics, with a very early salivary presence, followed by salivary and naso-pharyngeal mucosa co-presence, while 10 to 15 days after infection, saliva is likely to become negative, NPS positivity persisting for longer periods [16, 26, 27]. The window of saliva positive results might also explain our findings, in a series of subjects under surveillance, of SARS-CoV-2 being identified by NPS, not by saliva testing. The vast majority of these subjects underwent NPS for suspect symptoms, the onset of which occurred more than 1 week after the last, negative saliva test result. Moreover, these subjects adhered to the surveillance program less regularly than those identified by saliva testing. Overall, these results suggest that a level of efficacy even higher than that obtained by us could be reached when the salivary-based surveillance program is planned once a week. However, the application of such a schedule would not be affordable in view of the high number of tests to be performed by laboratories and related costs. These two limitations could be overcome by adopting pooling saliva analysis, which was demonstrated to be reliable in an elementary-to-high school real-world scenario, with a 24 samples pool [14]. In our study, a pooling strategy with a two samples pool was adopted, but this approach could be implemented in the future with more samples to expedite analyses. In this context, another strategy to be considered is antigen testing by highly automated laboratory platforms that combines high sensitivity, rapid results, and lower costs in terms of both reagents and personnel with respect to molecular testing [16, 28].

To verify whether our surveillance protocol was effective in limiting SARS-CoV-2 diffusion among participants, incidence in the surveillance cohort was compared with the incidence observed in a smaller cohort of employees who did not participate in the surveillance program, and with the incidence registered in the same period in the district of Padua and in the wider Veneto region. Although government databases might not be completely accurate especially during pick periods, the incidence among unsurveilled employees was equal to that of the Padua district and Veneto region, which were all more than twofold that recorded among surveyed employees. This result clearly demonstrates that saliva testing minimizes transmission in communities at risk, enabling early identification and further isolation of asymptomatic and pauci-symptomatic individuals, symptoms in the vast majority of subjects positive at saliva testing being absent or only mild.

The strength of our surveillance program was related to a highly organized collection program directly planned by the IT service of the University of Padua, to local logistic companies for sample transportation from the collection points to the laboratory, to pre-barcoded tubes, and to complete automation of sample processing that allowed a turn-around-time of less than 24 h. Moreover, the immediate communication of positive results from the laboratory to the Department of Preventive Medicine allowed a speedy process of isolation and contact tracing. We estimated the overall cost of the program considering in the computation all reagents and supply, logistic, and personnel costs. The overall costs of the program (55,998 salivary tests from October 2020 to June 2021) were 674,080 Euro, i.e., 12 euro per individual, a cost very close to the $12.5 reported by Mendoza et al. [14]. By adopting a policy of test pools larger than that used in this study, costs per test could be further reduced. It is important to bear in mind that cost estimation must take into account the financial saving incurred by reduced potential hospitalizations. Although it is somewhat difficult to estimate how many potential diseases were prevented, certainly the avoidance of any hospitalization allows a saving of from 30,000 to 70,000 euro [14], but above all saves lives.

In March 2021, the University of Padua started the vaccination program for its employees using the Chadox1/AZD1222 (Astra Zeneca) vaccine. On 11 March 2021, after warnings concerning this vaccine lot, we proceeded with BNT162b2 (Pfizer/BioNTech). We invited employees found to have contracted SARS-CoV-2 to perform antibody testing after 3 and 6 months. After 3 months, 3/104 subjects had negative serology, titers being correlated with age and Ct values, suggesting that a more advanced age and a higher initial viral load favors a more pronounced antibody production. Our finding of age-related antibody response is in agreement with data reported by Dorigatti et al. in their seroprevalence analysis conducted in Vo’ [29]. After 6 months, titers significantly declined in unvaccinated individuals this decline ranging from 26 to 72%. Vice versa, the significant increase found in antibody titers in vaccinated subjects was independent of age, but correlated with pre-vaccination levels of antibodies and vaccine type [29]. This finding should be cautiously evaluated and interpreted, since it does not necessarily mean that BNT162b2 induces more protection than Chadox1/AZD1222. We measured anti-RBD IgG with a validated assay, the results of which are correlated with a neutralizing activity [17]. In the presence of similar neutralizing activities, different diagnostic systems used to detect SARS-CoV-2 IgG might behave slightly differently [29, 30] and also might have a different sensitivity in detecting antibodies induced by different vaccines.

## Conclusions

In conclusion, our prospective surveillance study, performed in Italy on a large cohort of subjects during the second and third wave of the SARS-CoV-2 pandemic, reinforces the concept that large-scale saliva molecular testing and contact tracing in high-risk communities, such as schools and universities, maintains the SARS-CoV-2 incidence at low levels at a sustainable cost.

## Data Availability

The datasets used and/or analyzed during the current study are available from the corresponding author on reasonable request.

## Abbreviations

CLIA: Chemiluminescent immunoassay
Ct: Cycle threshold
IQR: Interquartile range
NPS: Naso-pharyngeal swab
QLAMP: Quantitative loop-mediated isothermal amplification
rRT-PCR: Real-time reverse transcription polymerase chain reaction
